# Sublethal Effects of CuO Nanoparticles on Mozambique Tilapia (*Oreochromis mossambicus*) Are Modulated by Environmental Salinity

**DOI:** 10.1371/journal.pone.0088723

**Published:** 2014-02-10

**Authors:** Fernando D. Villarreal, Gautom Kumar Das, Aamir Abid, Ian M. Kennedy, Dietmar Kültz

**Affiliations:** 1 Department of Animal Science, University of California-Davis, Davis, California, United States of America; 2 Department of Mechanical and Aerospace Engineering, University of California-Davis, Davis, California, United States of America; University of Kansas, United States of America

## Abstract

The increasing use of manufactured nanoparticles (NP) in different applications has triggered the need to understand their putative ecotoxicological effects in the environment. Copper oxide nanoparticles (CuO NP) are toxic, and induce oxidative stress and other pathophysiological conditions. The unique properties of NP can change depending on the characteristics of the media they are suspended in, altering the impact on their toxicity to aquatic organisms in different environments. Here, Mozambique tilapia (*O. mossambicus*) were exposed to flame synthesized CuO NP (0.5 and 5 mg·L^−1^) in two environmental contexts: (a) constant freshwater (FW) and (b) stepwise increase in environmental salinity (SW). Sublethal effects of CuO NP were monitored and used to dermine exposure endpoints. Fish exposed to 5 mg·L^−1^ CuO in SW showed an opercular ventilation rate increase, whereas fish exposed to 5 mg·L^−1^ in FW showed a milder response. Different effects of CuO NP on antioxidant enzyme activities, accumulation of transcripts for metal-responsive genes, GSH∶GSSG ratio, and Cu content in fish gill and liver also demonstrate that additive osmotic stress modulates CuO NP toxicity. We conclude that the toxicity of CuO NP depends on the particular environmental context and that salinity is an important factor for modulating NP toxicity in fish.

## Introduction

Manufactured nanoparticles (NP), with sizes ranging from 1 to 100 nm in two or three of its dimensions [1], have been increasingly employed in a broad range of applications including environmental remediation, cosmetics, biomedicine, material sciences and electronics [2], [3], [4]. The unique physical properties of NP are mainly attributed to their high surface to volume ratio, with a large proportion of the atoms being exposed on the surface compared to the bulk material [5]. In addition, factors including chemical composition, size, shape, solubility and aggregation are generally considered to be important parameters that determine the properties of NP [6]. Among manufactured NP, the metal and metal oxide NP are employed in a broad range of applications [7]. Copper (II) oxide (CuO) NP have been used to improve the efficacy of gas sensors, as high-transition-temperature superconductors, as catalysts, and as antimicrobial agents [8], [9], [10], [11], [12].

Cu-based NP, as other metal-based NP, could pose a biological hazard risk because of their ability to induce metabolic alkalosis, to release metal ions to the medium (or intracellularly), and to induce the intracellular generation of reactive oxygen species (ROS) [13], [14]. CuO NP showed higher toxicity than that of other metal-containing NP towards human epithelial lung cells [15], while they were found to display TC_50_ (toxic concentration to induce death of 50% of cells) ranging from 3 to 31.07 mg·L^−1^ in the macrophage cell line THP-1 [16]. Much of the toxicological studies of CuO NP to date has been focused on atmospheric or inhalation exposures. In contrast, very few studies have analyzed CuO NP effects on fish. One of them showed that, in FW carp, CuO NP were not acutely toxic, although growth was inhibited while CuO NP content in tissues increased over time, particularly in intestine, gills and liver [17]. Moreover, the authors observed a reduction in the tail beat frequency after 96 hours of exposure at 10 mg·L^−1^. Concentrations as low as 0.5 mg·L^−1^ of CuO NP interfered with hatching of FW zebrafish embryos [14]. CuO NP toxic effects have also been studied in other aquatic organisms [18], [19], [20].

The expanded use of manufactured NP in a growing number of products and the potential for release of these materials into the environment is raising concerns about their putative environmental toxicity [21]. Major technical limitations for assessing such ecotoxicological impact include the difficulty of isolating manufactured NP from complex samples as well as their diverse chemical behaviors (aggregation, dissolution, surface coatings) [3], [22]. Currently, data on the concentrations or chemical status of NP being released to the environment are scarce [23], [24]. As an alternative for actual measurements, mathematical models have been devised to determine the environmental concentration of manufactured NP. Such models suggest that concentrations in the order of ng·L^−1^ or µg·L^−1^ could already be present in certain environmental niches [25], [26], [27].

In general, the toxicity of metallic and metal oxide NP is attributed only partially to the release of ions resulting from dissolution. The properties of the surface of the NP are suspected to affect the induction of the toxicological response [6], [21], [28], [29]. Induction of oxidative stress, mediated by reactive oxygen species (ROS) generation at the NP surface, has been suggested to be a major culprit in the toxicological effects of multiple metal-based NP [6], [30], [31], [32]. Importantly, surface properties, particle aggregation status and dissolution attributes of NP are determined by the characteristics of the medium in which they are suspended, including pH, ionic strength or the presence of biomolecules. Therefore, the toxicity of NP towards aquatic organisms can be expected to depend on water quality parameters, including salinity [28].

Studies comparing the effects of NP in different aquatic environments are, however, scarce [33], [34]. Nonetheless, copper ions toxicity in *Fundulus heteroclitus* is dependent on the environmental salinity [35]. Additionally, several aquatic organisms showed an increased tolerance to copper ions with increasing salinity [36]. Similar trends were observed for nickel [37] and zinc [38] in *F. heteroclitus* and *Kryptolebias marmoratus*.

We have investigated the extent of toxicity of CuO NP on Mozambique tilapia (*Oreochromis mossambicus*) at different salinities by simulating a fresh water habitat and an estuarine habitat subject to tidally induced salinity fluctuations.

Tilapia are widely distributed in the wild in estuarine and inland habitats and extensively used in aquaculture [39]. Mozambique tilapia is a well-established model for investigating osmoregulatory mechanisms because of this species high capacity for adapting to a wide range of environmental salinities [40], [41], [42]. Furthermore, tilapia has been used for many toxicological studies, including studies investigating the effects of metals [43], [44] and metallic NP [45], [46].

Here, we address the question of whether environmental salinity alters biological responses of tilapia to CuO NP. The biological responses selected in this study as experimental endpoints include sublethal markers of toxicity such as behavioral stress and opercular ventilation rate (OVR), which have been widely used to monitor sublethal effects of diverse environmental stressors, including toxicants, on fish [47], [48], [49].

## Methods

### CuO NP synthesis

CuO NP were synthesized using a flame spray pyrolysis technique with forced jet atomizer (**Figure S1 in File S1**) similar to the method reported previously [50], [51]. This technique is well-suited for generating environmentally relevant metal oxide NP in high concentrations with control on particle size [52]. Copper nitrate salt was dissolved in ethanol to produce a 0.3 mM solution for use as the liquid precursor. The solution was sprayed into a 1∶1 hydrogen-nitrogen flame at 2.0 mL·min^−1^ with a syringe pump. A flow of argon was used as sheath at a rate of 10 L·min^−1^. The precursor droplets were pyrolyzed to form NP in the high temperature flame. Particles in the post-flame gases were extracted using a vacuum pump and collected in a baghouse (Filter Specialists Inc; Part # BNM05P4PA). The collected powder was washed several times with ultrapure water to remove any unreacted precursor from the NP. The NP were dried for further use.

### CuO NP characterization

#### Transmission Electron Microscopy (TEM)

TEM images were acquired using a Phillips CM-12 TEM operating at 120 kV. A drop of NP dispersion was put onto a formver carbon film supported on a 400 mesh copper grid (3 mm in diameter) and allowed to dry in air at room temperature. The grid was then mounted into the vacuum chamber for imaging.

#### Powder X-ray Diffraction (XRD)

Approximately 100 mg of powder sample was stirred gently to break up lumps. The powdery samples of the NP were then spread evenly onto a zero-background holder. Step-scan X-ray powder diffraction data were collected over the range of 2θ range of 20–85° on a Scintag powder x-ray diffractometer (XRD) with Cu Kα radiation (λ = 1.54056 Å, operated at 45 kV and 40 mA) with 4 mm divergence slit, 1 mm scattering slit, and 0.2 mm receiving slit. The scanning step size was 0.015° in 2θ with a counting time of 1 s per step.

#### Dynamic light scattering (DLS) and Zeta Potential (ZP) measurements

The hydrodynamic size distribution and state of agglomeration/dispersion of the CuO NP was analyzed by DLS using a Brookhaven ZetaPlus instrument (Brookhaven Instrument Inc. Holtsville, NY). The effective diameter particle size distributions were calculated by the 90Plus software (Brookhaven Instruments). The effective diameter corresponds to the first cumulant of the correlation curve and for multimodal distributions it is weighted roughly by scattering intensity. Samples (i.e. NP dispersed in water in fish tanks; concentration 0.5 or 5 mg·L^−1^, pH∼8.25) were taken from the tanks at every 24 h for analysis. Samples were transferred into a 4 ml cuvette without any filtration to determine the size and assess the extent of aggregation. The mean particle counts obtained in the measurements were 79 and 335 kcps for 0.5 and 5 mg·L^−1^ samples respectively. The ZP or overall surface charge of NP sampled from fresh water (FW) tanks was determined using the same ZetaPlus instrument (Brookhaven Instruments Corp.).

#### Brunauer–Emmett–Teller (BET) surface area measurements

The BET surface area measurements were carried out using N_2_ gas adsorption in an AUTOSORB-1 using an optimized protocol (Quantachrome Instruments, Boynton Beach, FL, USA). The samples were degassed at 120°C for 12 h prior to the adsorption-desorption cycle.

All data of NP characterization (TEM, DLS, ZP and ICP-MS can be found in **Table S1 in File S1**).

### Fish acclimation and care

Tilapia maintenance and the experimental conditions were subject to approval by the UC Davis Institutional Animal Care and Use Committee, under protocols #16604 and #16609. Throughout experiments, all efforts were made to minimize animal suffering. Laboratory-raised Mozambique tilapia (*Oreochromis mossambicus*) were maintained in large closed circulation aquaria using de-chlorinated tap water (pH 8.25, total hardness 155.5 mg·L^−1^) at 26°C±1°C and 12 h∶12 h photoperiod. Two weeks prior to exposure, twelve animals (10–12 months old) were transferred to experimental tanks with 16 L of freshwater for pre-acclimation, and kept under those conditions until treatment was started. Fish were fed daily with trout pellet diet (Silvercup SCD 2.0 mm) at approximately 1% of fish body weight. For all treatments, 10% of the water was changed every day for all tanks. For this, 1.6 L of the tank water was removed, and 1.6 L of water supplemented with appropriate NP concentration and salt as required (see below) was added. The salinity was monitored with a refractometer before and after the daily water change. A group of tanks (denoted as FW) were kept at freshwater conditions (tap water, with a salinity of 0–0.1 parts per thousand, ppt, corresponding to g·L^−1^). On the other hand, tap water supplemented with sea salt (Instant Ocean aquarium seawater salt, Vancouver, USA) was used for a second group of tanks (SW), at amounts required to daily increase salinity by 7 ppt/day, except for the first day when the change was from 0 to 3 ppt. For both FW and SW groups, animals were exposed to 0, 0.5 and 5 mg·L^−1^ of CuO NP through addition of a concentrated CuO stock solution (10 g·L^−1^ in MilliQ water) that was probe-sonicated (Branson Digital Sonifier 250, Branson Ultrasonics Corporation) with the maximum output of 200 W, and with a frequency of 20 kHz, for 5 minutes before addition to the tanks. To avoid any temperature rise in the solution, the sample was sonicated in cycles of 20 s at 10% of maximum power, followed by 20 s rest. After water changes, manual stirring was performed for 20 seconds to ensure dispersion of the NP in the tanks. The salinity was confirmed with a refractometer before and after the daily water change. FW0 treatment is the handling control, where the fish were subjected to the same interaction than the animals in the rest of the tanks (water change, manual stirring, observation), but without salinity increase nor NP addition.

The animals were observed for signs of stress on a daily basis. The experimental exposure endpoint was established as the time when more than 50% of the animals in the tanks showed behavioral signs of stress (e.g., buoyancy difficulties, swimming abnormalities, lack of feeding) or when the mean opercular ventilation rate (OVR) was 25% higher than to the untreated FW control for two consecutive days. The animals were sacrificed; the gill arches and liver were collected. The gill arches were rinsed with cold PBS and gill epithelium was gently scraped off from the cartilage and collected. Tissues were frozen in liquid nitrogen and stored at −80°C until used.

Additionally, we performed a similar experiment in were all experimental exposure conditions were replicated (aeration, stirring, water changes), except that no fish were included in the tanks.

### Cu concentration determination by ICP-MS

CuO NP samples from experimental tanks with or without fish (taken before of the daily water change except for time = 0, where samples were taken immediately after addition of NP to the tank) were used to determine total Cu content and to measure soluble copper. To take representative samples from the tanks, we stirred the tanks for 20 sec before taking the sample. Additionally, each sample (10 ml final volume) was taken by pipetting 5× (2 ml) aliquots, which were combined, from different points in each tank. Samples were first digested with 70% HNO_3_ (trace metal grade concentrated nitric acid, Optima, Fisher Scientific) at 70°C for 2 h. Then the samples were diluted to 6% HNO_3_ with MilliQ water and analyzed by inductively coupled plasma mass spectrometry (ICP-MS) for determination of the total Cu content. To determine the concentration of soluble copper ions, CuO NP samples from experimental tanks were loaded in dialysis bags (MWCO 3,500 Da, Spectrum Spectra/Por 3 RC) and dialyzed overnight against water used for the fish tanks. This method has been shown to be suitable to separate solubilized Cu^2+^ ions and Cu as particulate phase [53]. The dialyzed water was digested in 70% HNO_3_ and diluted to 6% HNO_3_ similar to other samples.

Tissue samples were treated in 70% HNO_3_ (Optima, Fisher Scientific) and hydrogen peroxide (34–37%, Technical, Fisher Scientific) for elemental analysis. At first, wet tissues were dried by overnight incubation in an oven at 110°C. Dry weight was recorded and tissues were then digested in 70% HNO_3_ at 80°C for 4 h, followed by hydrogen peroxide digestion overnight (70°C). Tissue samples were diluted with MilliQ water to an acid concentration of 6%.

The concentration of elemental copper was quantified using ICP-MS at the Interdisciplinary Center for Plasma Mass Spectrometry at UC Davis. The instrument level of detection (LOD) was 0.019 parts per trillion and the BEC (background equivalent concentrations) were 0.125 parts per trillion for Cu.

For statistical analysis of Cu content in tissues, data (in ng·mg^−1^ dry weight) were transformed to logarithms since the variances proved not to be homogeneous as indicated by Levene's test. The transformed data were then analyzed by one way ANOVA followed by the Tukey post-hoc test.

### Enzyme activity assays

Tissues were homogenized in 1.5-mL microcentrifuge tubes after the addition of 5 µL of homogenization buffer (50 mM potassium phosphate pH 7.4, 0.9% NaCl, 0.1% glucose, 1X protease inhibitor cocktail cOmplet Mini, Roche) per mg of tissue. Homogenates were centrifuged at 14000 rpm and 4°C for 20 min; the supernatant protein concentration was determined by BCA assay (Pierce, USA). Catalase (CAT), superoxide dismutase (SOD) and glutathione reductase (GR) enzymatic activity were determined using specific assay kits (Cayman Chemical, USA), following vendor instructions. Data were analyzed by One way ANOVA followed by Tukey's post-hoc test to compare all the means and every treatment pair separatedly.

### Oxidative status (GSH/GSSH ratio)

The oxidized (GSSG) and total (GSH+GSSG) glutathione levels were determined by the reaction with Ellman's reagent 5,5′-dithio-*bis*-2-nitrobenzoic acid, DTNB [54]. Tissues were homogenized in 10 volumes of 5% sulfosalicylic acid (SSA) or 5% SSA plus 3 mM of 1-Methyl-2-vinylpyridinium triflate (M2VP, Sigma), for total or oxidized glutathione respectively [55]. After 30 min of incubation on ice, samples were centrifuged (14000 rpm, 4°C, 20 min), and supernatant transferred to new tubes. Samples were neutralized to pH 7–7.2 by addition of neutralization buffer (2.125 M Na_2_CO_3_) and combined with assay mixture: 100 mM potassium phosphate pH 7.0 and 1 mM EDTA, 0.13 U·mL^−1^ glutathione reductase (Sigma), 0.04 mg·mL^−1^ NADPH (Fisher) and 0.0325 mg·mL^−1^ DTNB (Sigma). Absorbance at 405 nm was determined at 25 minutes using a Spectrafluor Plus microtiter plate reader (Tecan). Oxidized GSSG (Sigma) was used to build a standard curve. Data were analyzed by One way ANOVA followed by Tukey's post-hoc test.

### Quantitative PCR (qPCR)

Total RNA was extracted from tissues using TRIzol reagent (Invitrogen, Life Technologies), and subsequently treated with TURBO DNase (Ambion, Life Technologies). cDNA was produced from 1 µg of RNA using Random Hexamers and ImProm II reverse transcriptase (Promega). Reactions were performed in MicroAmp Fast Optical 96-well plate thermal cycling plates (Applied Biosystems, Life Technologies) using Fast SYBR Green Master Mix (Applied Biosystems, Life Technologies) and primer pairs designed with Primer-BLAST [56]: *Metallothionein* (5′-GCGAGTGCGCCAAGACTGGAA-3′ and 5′-CAGCCGGATGGGCAGCAGTC-3′), *Cytochrome P4501A* (5′-TGTCACCGAGCACTATGCCACCT-3′ and 5′-TCCAGCTTTCTGTCCTCGCAGTG-3′), and *β-actin* (5′-CCACAGCCGAGAGGGAAAT-3′) and (5′-CCCATCTCCTGCTCGAAGT-3′). Reactions were performed in a 7500 Fast Real-Time PCR system (Applied Biosystems), using the following cycling conditions: 1 cycle at 95°C/20 s followed by 40 cycles of 95°C/3 s and 60°C/30 s. The Pfaffl method [57] was used to calculate the ratio of mRNA expression in treated fish relative to the fresh water handling controls, using *β-actin* as a reference transcript for data normalization. Data were corrected for individual PCR efficiencies (LinRegPCR software [58]). Data were then analyzed using ANOVA on ranks (Kruskal-Wallis's test) and Wilcoxon signed rank statistic, given that these data were non-parametric.

## Results

### CuO NP are highly crystalline and monoclinic

**Figure 1:**
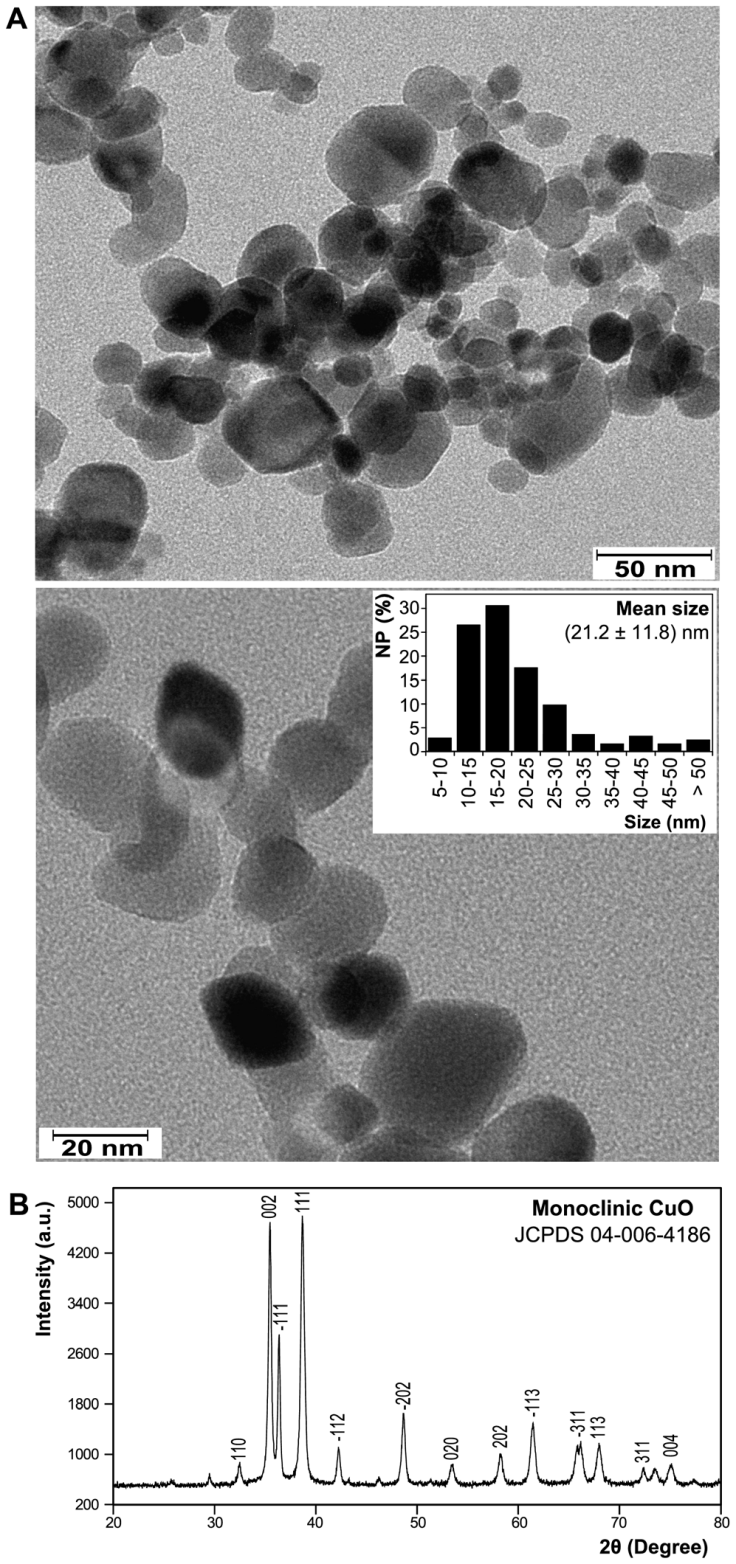
Characterization of CuO NP used in the present study. (A) Representative transmission electron microscopy (TEM) images of the CuO NP at two magnifications. The polydisperse size distribution of CuO NP (n = 245) determined from TEM images is shown in the insert. The average size of the NP is shown (mean ± *SD*). (B) X-Ray diffraction (XRD) pattern of the NP. The X, and Y axes of the XRD represents the angles (2θ) of incident X-ray beam and the corresponding diffraction peak intensity.

The flame-synthesized NP were characterized by TEM. **Figure 1A** shows typical TEM micrographs of the CuO NP. Quantitative analysis of these micrographs shows that the synthesized CuO NP were polydisperse with a mean size of 21.2 nm and a standard deviation of 11.8 nm (n = 245); 77.4% of the NP were smaller than 25 nm (**Figure 1A** **, insert**). The darker appearance of some NP is mainly attributed to different NP thickness and orientation when dried on the TEM grid, for which electrons scatter incoherently and produce differential contrast. The crystallinity and phase of the NP were determined by powder X-Ray diffraction. The XRD pattern (**Figure 1B**) confirms that the NP are highly crystalline and consist of monoclinic CuO (JCPDS 04-006-4186). The XRD pattern of NP after probe sonication was also analyzed (**Figure S2 in File S1**) to investigate any possible physicochemical changes due to sonication. The XRD patterns of the NP before and after sonication are identical which confirms retention of their same phase and crystallinity.

**Figure 2:**
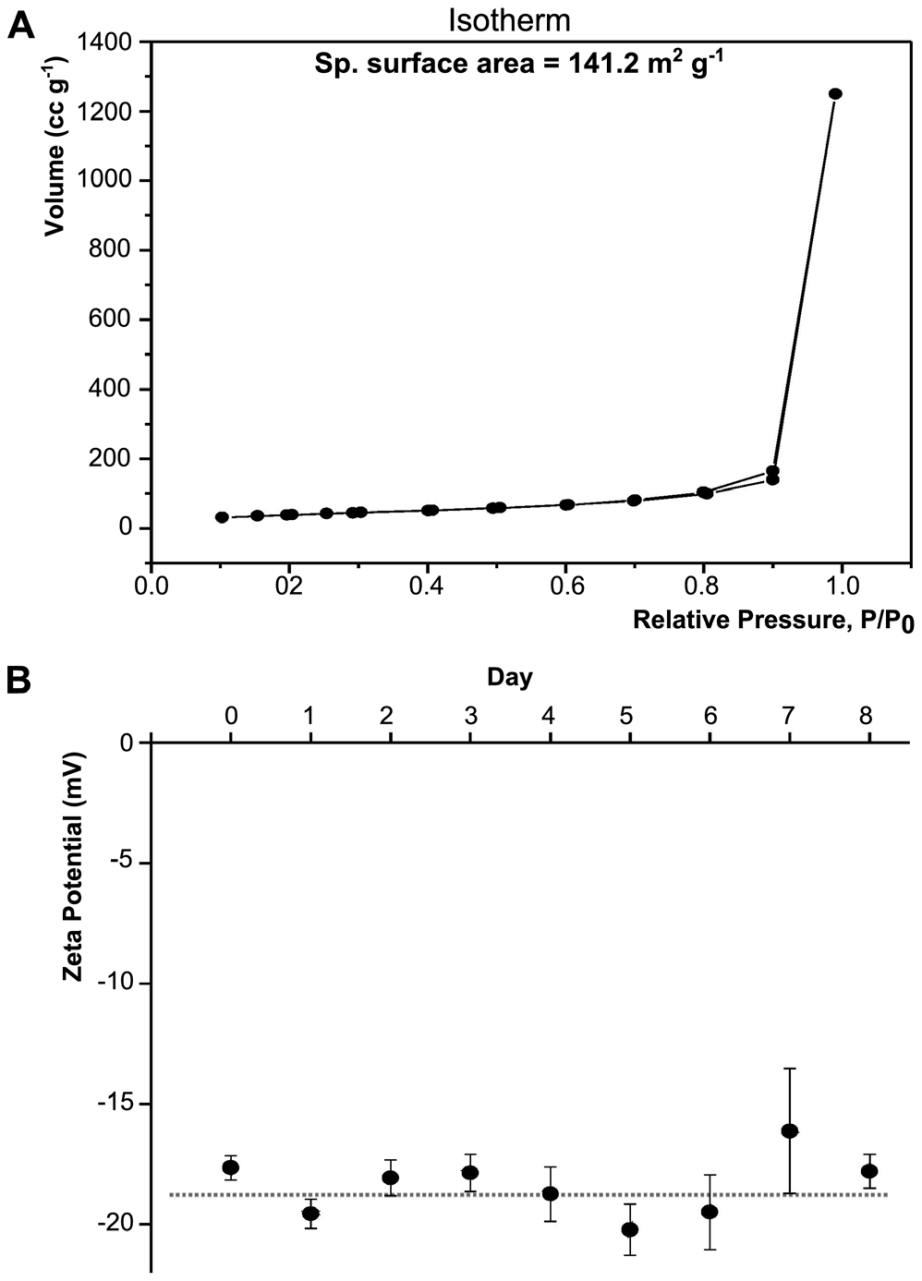
BET and Zeta Potential of CuO NP. (**A**) BET isotherm of the CuO NP (powder). (**B**) Zeta Potential (ZP) of the NP sample taken from FW5 tanks over the time. Day 0 represents the ZP of the NP right after mixing to the fresh water of the fish tank. The ZP of the NP remained fairly constant though out the experiments. The dotted line showed as a guide.

The surface area of the CuO NP was found to be 141.2 m^2^·g^−1^ (the isotherm is presented in **Figure 2A**). **Figure 2B** and **Figure S3 in File S1** show the Zeta Potential (ZP) of the NP samples taken from fresh water tanks over an 8 day period. The magnitude of the ZP can be taken as one of the parameters to understand the colloidal stability of the NP [59]. NP with ZP values greater than +30 mV or less then −30 mV typically have a high degree of stability in suspension [59]. We analyzed the ZP of the NP dispersed in fresh water only (**Figure 2B**); the ZP was seen to remain constant over time, which indicates good colloidal stability of the NP. However, it was not possible to obtain the ZP of NP dispersed in saline water (i.e. at dissolved salt concentration ranging from 0.04–0.6 M NaCl). As per the DLVO theory (Derjaguin, Landau, Verwey and Overbeek), the agglomeration and stability of particle dispersions are determined by the sum of the attractive and repulsive forces among individual particles. The attraction within the particles is mainly due to the van der Waals force while the electrical double layer surrounding each particle offers electrostatic repulsive force among particles. The zeta potential and the thickness of the electrical double layer are two important parameters of the electrical double layer and an increase in either will result in an increase in the electrostatic repulsive interaction. The thickness of electrical double layer is a function of solution ionic strength, with an increase in ionic strength leading to a decrease in double layer thickness. For ZP measurement, at a salt concentration of 0.001 M the dispersions are generally stable as the electrostatic repulsive forces dominate over the attractive forces. However, at an increased concentration of 0.01 M NaCl or higher, the attractive forces among the particles started to dominate over the repulsive forces (due to compression of the electrical double layer) resulting an unstable, highly agglomerated dispersion. The concentration we used in our study was 0.04 M at day 1 and stepwise increased to 0.6 M NaCl at day 8. Therefore, any attempt to measure ZP of the solutions at these salt concentrations resulted in visible particle agglomeration and erroneous results. This observation is consistent with previous studies such as those from Jiang *et al.* [60] and Brant *et al.* [61].

### CuO NP exposure affects salinity-stressed fish differently than FW controls

**Figure 3:**
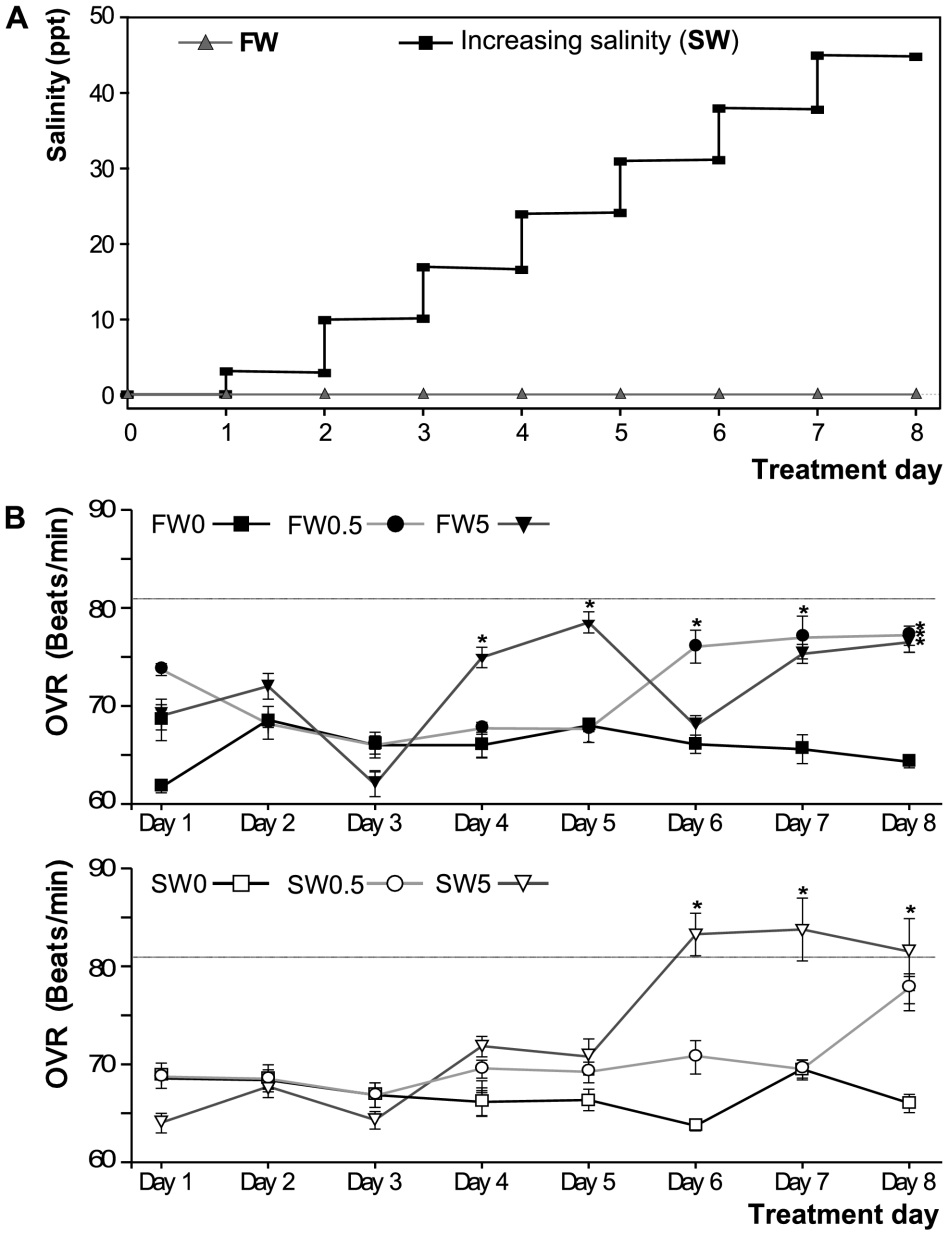
Experimental conditions during CuO NP exposure. (**A**) Salinity changes regime for FW and SW tanks. The FW tanks were kept in constant freshwater (0.1 ppt, gray triangles), while in SW tanks, salinity was increased in a stepwise manner every day (black squares), by addition of increasing amounts of sea salt. Salinity increase steps were of 7 ppt/day, except for the first day when the increase was of 3 ppt. (**B**) Opercular ventilation rate (OVR) in experimental tanks, measured as opercular beats/minute and expressed as the daily mean per tank (n = 7). The dotted line represent the 25% increment selected as experimental end point. Asterisks denote significant differences relative to the FW0 per day (*P*<0.05). Top: FW0, fresh water; FW0.5, FW plus 0.5 mg·L^−1^ CuO; FW5, FW plus 5 mg·L^−1^ CuO; Bottom: SW0, increasing salinity; SW0.5, SW plus 0.5 mg·L^−1^ CuO; SW5, SW plus 5 mg·L^−1^ CuO. *n*×tank = 7.

**Figure 3A** shows salinity levels in tanks with a control fresh-water environment exposed fish and animals that were challenged with stepwise increasing salinity. Fish from both groups (salinity challenge -SW- and freshwater -FW-) were exposed to 0, 0.5 or 5 mg·L^−1^ CuO NP. Opercular ventilation rate (OVR) means for each experimental group ranged from 62 to 84 beats/min (**Figure 3B**). An increase greater than 25% in OVR (compared to FW0 control) was observed after 6 days of exposure to increasing salinity (at 31 ppt), in SW5 (26.0%±3.3%). This increase was sustained at day 7 (salinity = 38 ppt) and day 8 (salinity = 45 ppt) (**Figure 3B** **, dotted line**). Therefore, the exposure was stopped at day 8. By day 8, the mean OVR was also significantly increased in all the other NP treatments, including FW0.5 and FW5. In addition, the behavioral signs of stress observed in the treatment groups were mild and pertained to only a minority of fish in a particular treatment group at any given time (**Table 1**).

**Table 1:** Stress signs on fish observed during the CuO NPs exposure.

Tank	Day	Stress sign	*n*	%	Intensity
**FW0.5**	6	Loss of buoyancy/swimming control	2	16.7	Mild
**FW5**	5	Late response to feeding	4	33.3	Mild
	6	Loss of buoyancy/swimming control	1	8.3	Mild
	7	Late response to feeding	5	41.7	Mild
**SW0**	5	Loss of buoyancy/swimming control	1	8.3	Mild
**SW0.5**	5	Loss of buoyancy/swimming control	2	16.7	Mild
**SW5**	5	Loss of buoyancy/swimming control	2	16.7	Mild
	7	Late response to feeding	4	33.3	Mild

*n*: number of animals per tank showing the stress sign (the percentage of stressed animals is also shown). *Intensity*: represents at what extent the observed stress signs affected the normal behavior of the animals.

### CuO NP concentration and size at different salinities

**Figure 4:**
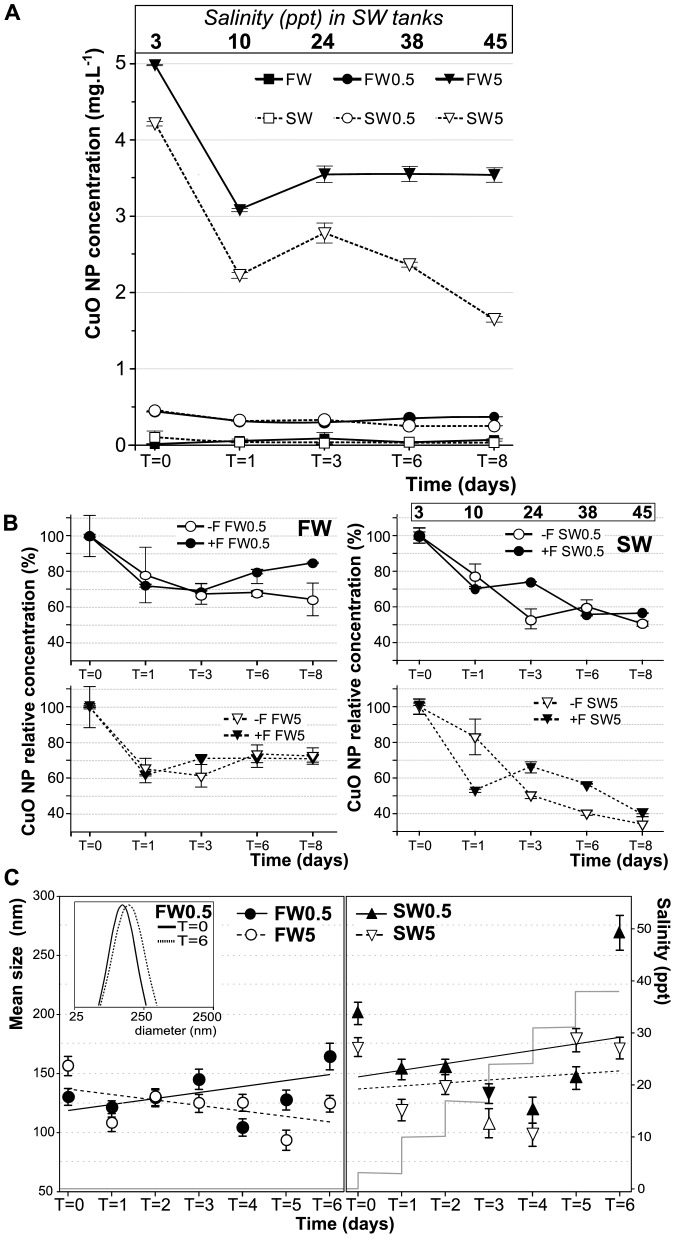
CuO NP behavior in experimental tanks. (**A**) CuO NP concentration during exposure in water samples from the experimental tanks (showed mean ± *SD*). (**B**) Time course of CuO NP concentration (mean % ± *SD*) relative to T = 0 for in FW0.5 and FW5 (left), SW0.5 and SW5 (right) treatment groups with fish (+F, black circles) and without fish (−F, open circles). Salinity (in ppt) at each time point is depicted for SW treatments. (**C**) CuO NP size in experimental FW (*left*) and SW (*right*) tanks analyzed by DLS. The inset depicts an example of the log-normal distribution data for the FW0.5 tank at days 0 and 6. Linear trend lines for each treatment are shown. Additionally, tank salinity in relation to time of exposure is shown (light gray lines). FW0, fresh water; FW0.5, FW plus 0.5 mg·L^−1^ CuO; FW5, FW plus 5 mg·L^−1^ CuO; SW0, increasing salinity; SW0.5, SW plus 0.5 mg·L^−1^ CuO; SW5, SW plus 5 mg·L^−1^ CuO. T = 0 corresponds to the sample taken immediately after CuO NP addition and the initial salinity increase. T = #: sample taken after # days of CuO exposure.

NP concentration in water samples taken from each group was below the expected value (based on mass added to a known volume of water), even at the beginning of the experiment (T = 0), particularly for SW tanks (**Figure 4A**). Within the first day of exposure (T = 1), the suspended CuO NP concentration decreased from 71% to 52% in all treatments compared to the CuO levels at T = 0 (**Figure 4B**). This trend was sustained over time. For instance, the suspended CuO NP concentrations in SW0.5 and SW5 were 73.4% and 65.8% at T = 3, 54.7% and 55.9% at T = 6, and 55.4% and 38.9% at T = 8, respectively (**Figure 4B**, black symbols). A comparable change in NP concentration over time was observed in absence of fish (**Figure 4B**, white symbols). No additional dissolved (soluble) Cu ions were detected after dialysis of samples taken from any of the NP experimental tanks (data not shown) when compared to the water from tanks without NP. Therefore, the change in NP concentration might be due to NP aggregation, or adsorption to glass surfaces in the tanks. However, visual inspection of the tanks did not reveal any sedimented material, which can be attributed to the formation of sedimentation particles with a size not large enough to be visible by the naked eye.

The state of aggregation of the CuO NP that remained suspended during exposure was determined by measuring the hydrodynamic size of the particles by DLS (**Figure 4C**). The hydrodynamic size of suspended CuO NP was larger than that observed by TEM, ranging from 93.54 nm±8.52 in FW5 at day 5 to 269.19 nm±14.7 in SW0.5 at day 6. The time course of CuO NP hydrodynamic size was similar for both concentrations in FW and for both concentrations in SW, as can be seen by the trend lines in **Figure 4C**. In FW0.5, FW5, and SW5 the hydrodynamic size fluctuated within a narrow range. In SW0.5, however, there was a significant increase in hydrodynamic size at T = 6 (38 ppt, corresponding approximately to ocean salinity), suggesting that some CuO NP aggregation had occurred under those conditions. This effect may be due to a bridging of NP via ionic bonds to form NP-M^+^-NP (where M^+^ is the salt cation, Na^+^ in the case of seawater that promotes NP aggregation [62]. In addition, divalent cations such as Mg^2+^ and Ca^2+^ (present in the seawater salt) have shown to induce aggregation of NP [63]. The concentration of monovalent cations (Na^+^ - K^+^) in sea salt is approximately 8 times higher than Mg^2+^ and Ca^2+^ concentrations combined [64].

### CuO NP exposure alters the activity of antioxidant enzymes in liver and gills

**Figure 5:**
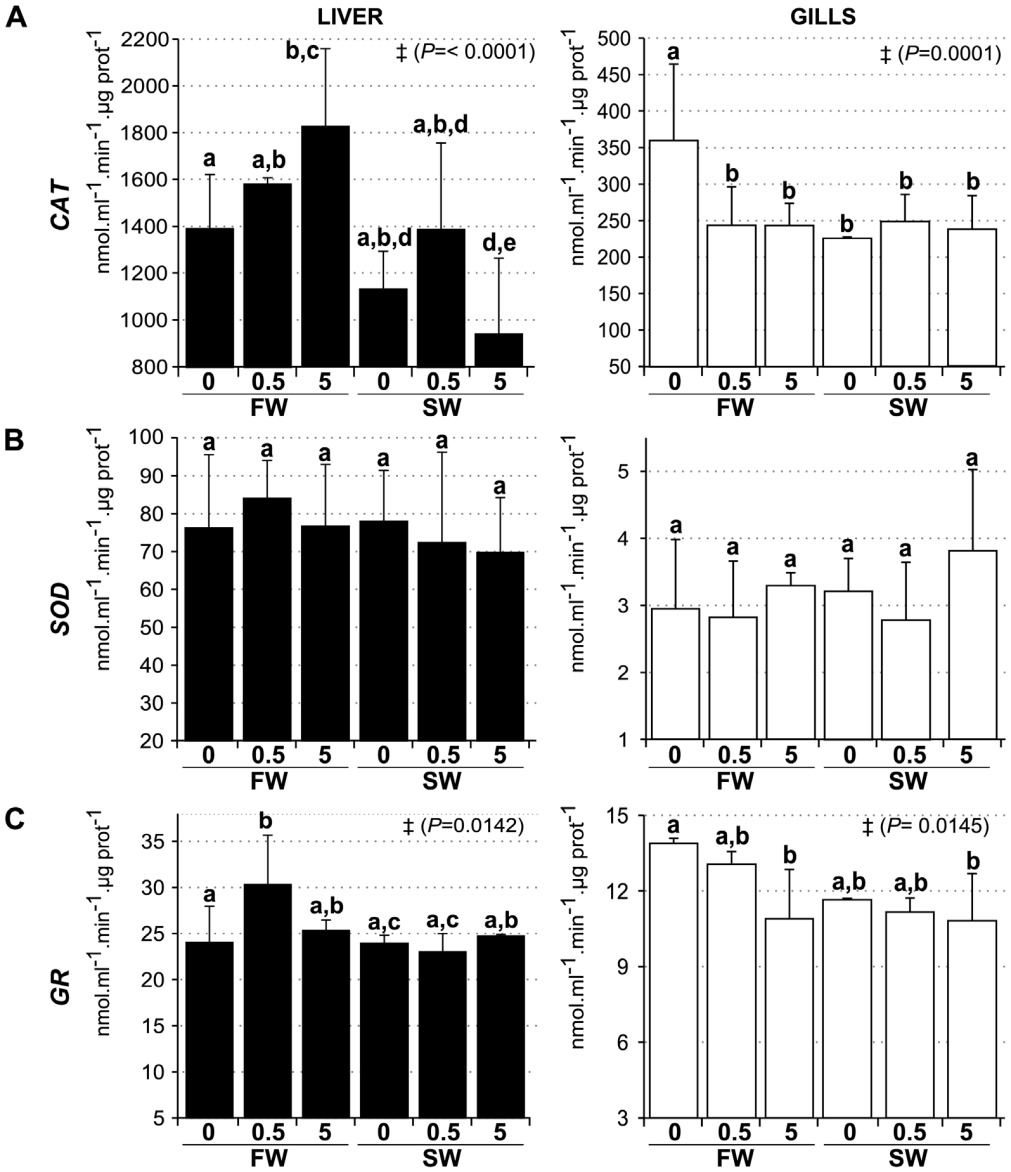
Analysis of enzymatic activity. (**A**) Catalase, (**B**) Superoxide Dismutase and (**C**) Glutathione Reductase were measured in homogenates from liver (black bars) and gills (white bars) after exposure to CuO NP in different environmental salinities. FW0, fresh water; FW0.5, FW plus 0.5 mg·L^−1^ CuO; FW5, FW plus 5 mg·L^−1^ CuO; SW0, increasing salinity; SW0.5, SW plus 0.5 mg·L^−1^ CuO; SW5, SW plus 5 mg·L^−1^ CuO. Activity is expressed as mean nmol.ml^−1^.min^−1^.µg prot^−1^ ± *SEM*. Groups with significant different means by Anova analysis are shown with ‡ (*P* value). Letters denote groups showing non significant differences by Tukey's post-test (*P*<0.05). *n*×treatment = 5.

To determine whether CuO NP can trigger an oxidative stress response in fish tissues, we measured the activity of three antioxidant enzymes in gills and liver after CuO NP exposure of fish (**Figure 5**). In both liver and gills, CAT (**Figure 5A**) and GR (**Figure 5C**) showed significant activity changes, whereas no changes in the total SOD activity were observed in response to CuO NP exposure (**Figure 5B**). Liver CAT activity decreased significantly in SW5 relative to FW0 controls. In contrast, CAT activity increased significantly in FW5 compared to FW0 controls indicating that the response to CuO NP is context-dependent and modulated by environmental salinity. GR activity increased significantly at FW0.5 compared to FW0 in liver, and decreased at both FW5 and SW5 compared to FW0 in gills. Additionally, gills CAT activity decreased significantly in response to salinity challenge and CuO exposure but there was no additive effect of both treatments (**Figure 5C**).

**Figure 6:**
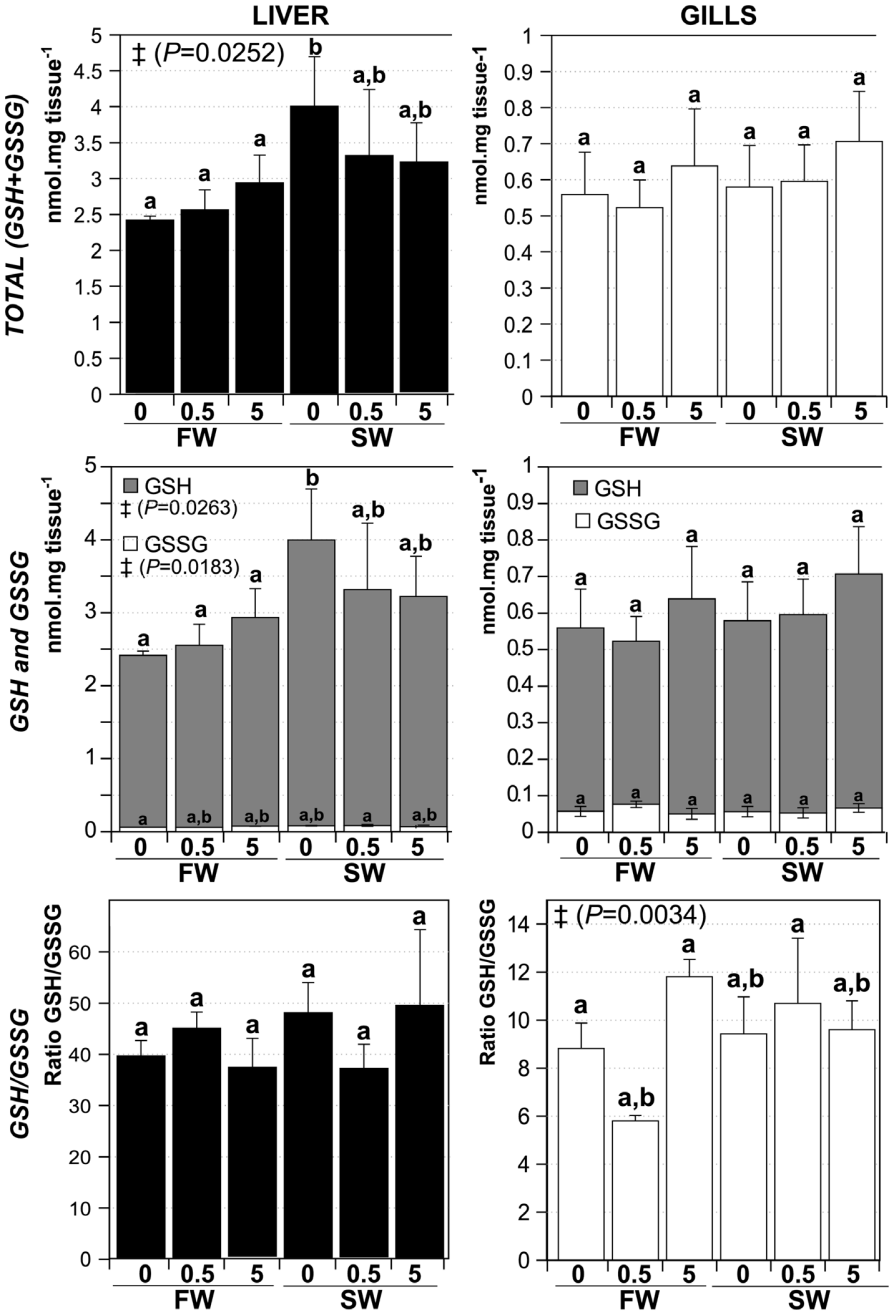
Glutathione levels in livers and gills of CuO NP exposed fish in different salinities. Total glutathione (GSH+GSSG, top panel); Oxidized glutathione (GSSG) and reduced glutathione (GSH) levels (center panel). The ratio GSH/GSSH is shown in bottom panel. FW0, fresh water; FW0.5, FW plus 0.5 mg·L^−1^ CuO; FW5, FW plus 5 mg·L^−1^ CuO; SW0, increasing salinity; SW0.5, SW plus 0.5 mg·L^−1^ CuO; SW5, SW plus 5 mg·L^−1^ CuO. Expressed as mean nmol.mg tissue^−1^ ± *SD*. Groups with significant different means by Anova analysis are shown with ‡ (*P* value). Letters denote groups showing non significant differences by Tukey's post-test (*P*<0.05). *n*×treatment = 5.

Another marker for oxidative stress in response to toxicants is the amount of glutathione and the ratio of reduced (GSH) versus oxidized (GSSG) glutathione [65], [66]. Therefore, total (GSH+GSSG) and oxidized (GSSG) glutathione concentration were measured with the intent to estimate GSH levels and calculate the ratio GSH/GSSG in gills and liver of fish exposed to CuO NP (**Figure 6**). Total GSH+GSSG levels increased significantly in SW0 liver compared to FW0 but in none of the other treatments (correlated with an increase in GSH levels), although all SW groups showed the same trend. The only significant change with respect to the GSH/GSSG ratio was a decrease in the ratio in gills following the FW0.5 treatment.

### Metal-responsive genes are induced by CuO NP enzymes

**Figure 7:**
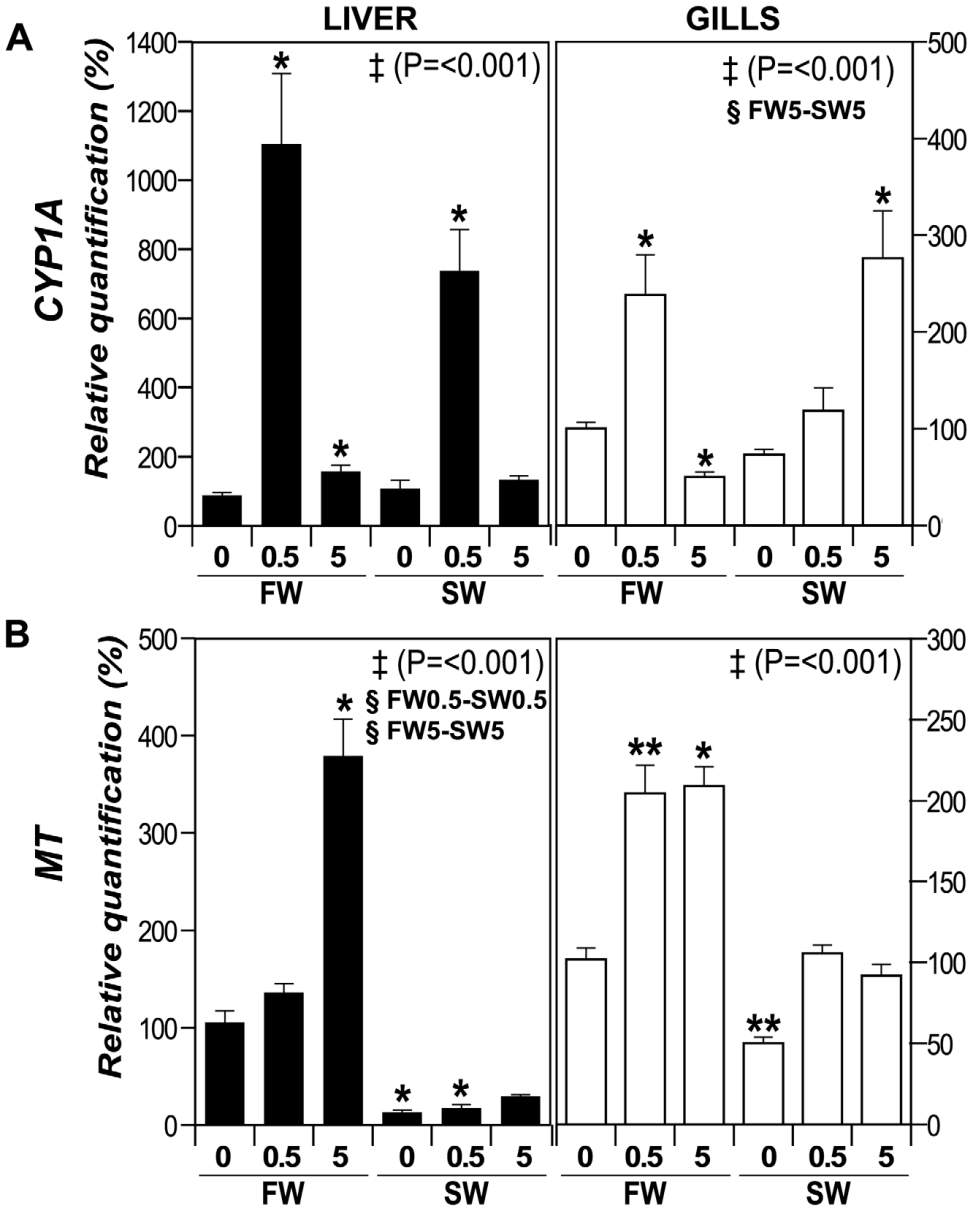
Expression of metal-responsive genes. Transcripts levels analysis by qPCR in liver (black bars) and gills (white bars). Transcript accumulation of (**A**) *Cytochrome P450 1A* (*CYP1A*), and (**B**) *Metallothionein* (*MT*). The genes were analyzed by qPCR in gills and liver, and normalized as function of the levels of endogenous control *β-Actin* gene. FW0, fresh water; FW0.5, FW plus 0.5 mg·L^−1^ CuO; FW5, FW plus 5 mg·L^−1^ CuO; SW0, increasing salinity; SW0.5, SW plus 0.5 mg·L^−1^ CuO; SW5, SW plus 5 mg·L^−1^ CuO. Results expressed as percentage of relative quantification. Significant different means by Anova on ranks analysis are shown with ‡ (*P* value), and significant differences between FW-SW at the same CuO NP concentration (Dunn's post test, *P*<0.05) are shown with §. Asterisks represent significant difference compared to the respective FW0 control by Wilcoxon Signed Rank test (*, *P*<0.05; ** *P*<0.025). *n*×treatment = 5.

We analyzed the expression of two genes involved in detoxification against metals, such as *Metallothionein* (*MT*) and *Cytochrome P450 1A (CYP1A)*. MTs are ubiquitous, low-molecular weight, Cys-rich proteins, capable of binding heavy metals (including Cu), which typically show an up-regulated gene expression when intracellular metal concentrations increases [67]. CYP1A, belonging to the Cytochrome P450 family of proteins, is involved in detoxification of many toxic compounds [68] and is encoded by a metal-responsive gene in fish [69], [70], [71]. The *CYP1A* promoter region in fish contains putative metal-response elements, MREs [72] and MT induction has been shown to occur after ionic Cu exposure [73]. mRNA abundances were in general strongly affected by CuO NP in both liver and gills, when compared to the FW0 control (**Figure 7**, asterisks). For example, *CYP1A* expression was greatly induced in liver after treatment with 0.5 mg·L^−1^ CuO NP in both FW and SW (1104% and 738%, respectively), and it was also slightly induced in FW5 (**Figure 7A**). In gills, *CYP1A* was increased to 238% in FW0.5 and 276% in SW5, whereas it was repressed to 50.1% in FW5. Salinity alone did not affect *CYP1A* expression as it can be seen in SW0 treatment in liver compared to FW0, although in gills we observed an increased expression of *CYP1A* in SW5 when compared to FW5 (§ symbol).

In liver *MT* (**Figure 7B**) showed a 379% increase in FW5, while a reduction in *MT* transcript levels was observed in SW0 (12.8%) and SW0.5 (17%) when compared to FW0. In gills, *MT* transcript accumulated to 204% and 209% for FW0.5 and FW5, correspondingly, while it was reduced to 49.9% in SW0, always relative to FW0. A significant decrease of *MT* expression in liver was observed in SW-NP treatments when compared to the FW treatments (§ symbol), although in SW5 the levels are comparable to FW0 (so increased relative to SW0 and SW0.5). Therefore, *CYP1A* levels are in general higher in the liver in response to CuO NP exposure. In contrast, *MT* was generally induced in FW treatments and repressed in most SW treatments (when compared to FW0) in both liver and gills suggesting a modulation of CuO NP effects by environmental salinity.

### Copper content in tissues exposed to CuO NP

**Figure 8:**
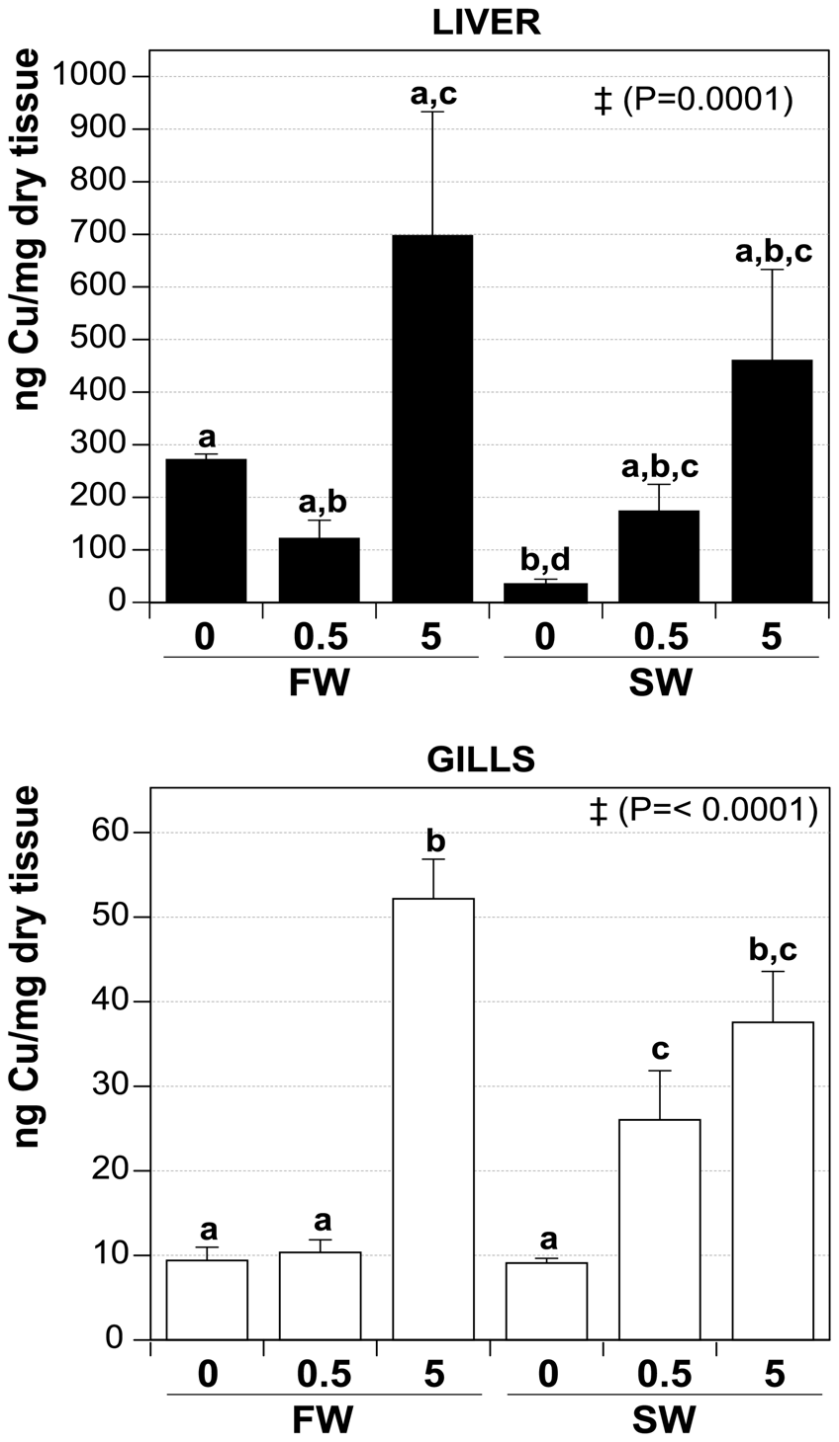
Cu-content of liver and gills after CuO NP exposure at different salinities. Values (ng of Cu per mg of dry weight) are depicted as mean ± SEM. FW0, fresh water; FW0.5, FW plus 0.5 mg·L^−1^ CuO; FW5, FW plus 5 mg·L^−1^ CuO; SW0, increasing salinity; SW0.5, SW plus 0.5 mg·L^−1^ CuO; SW5, SW plus 5 mg·L^−1^ CuO. Significant different means by Anova analysis are shown with ‡ (*P* value). Letters denote groups showing non significant differences by Tukey's post-test (*P*<0.05). *n*×treatment = 5.

Cu concentration was determined by ICP-MS in liver and gills of animals exposed to CuO NP (**Figure 8**). In both tissues there was a consistent trend towards an increase in Cu content with increased concentration of Cu NP in the environment. This trend was also true for the liver, although the significance threshold for FW treatments was not exceeded in any case when CuO treatments were compared to the FW0. However, the content of Cu in SW0 was inferior when compared to the FW0 and to FW5, SW0.5 and SW5, suggesting an accumulation of Cu in SW treatments (when compared to the SW0 treatment). In gills, Cu accumulated significantly in both SW treatments, but not in FW at 0.5 mg·L^−1^ CuO when compared to FW0. Additionally, accumulation of Cu in SW0.5 was significantly different to FW0, FW0.5 and SW0, while FW5 and SW5 treatments were higher when compared to their respective non NP treatment, although it was comparable between them.

These data show that even when Cu is effectively accumulated in both tissues when fish are exposed to CuO, the degree of this accumulation is to some extent dependent on the environmental salinity, since in SW treatments, Cu accumulated effectively followed NP treatment compared to the SW0 in both tissues.

## Discussion

Salinity changes represent a relevant variable to test if NP toxicity in fish is affected by exposure to different environmental conditions. The osmotic stress induced by exposing fish to environmental salinity fluctuations has been well-characterized, from sensing mechanisms in fish [74] to the study of strategies developed by the organism to cope and adapt to the osmotic changes [75], [76]. Therefore, the use of hyperosmotic stress in tilapia its a useful tool to understand how NP may have effects on fish under different environmental conditions. In laboratory conditions, gradual (stepwise) acclimation to salinity changes has been largely used to model osmotic stress in different fish species, allowing a better assessment of chronic effects of osmotic stress [77]. Salinity changes in the environment can be seasonal, diurnal and spatial, depending on factors such as rainfall, tides, runoffs and evaporation [78]. Several tilapia species have been described in FW to hypersaline habitat conditions [41]. Their high salinity tolerance may have evolved as a result of strong selection pressure associated with frequent seasonal droughts and intermittent flooding events in areas characterized by salt-rich bedrock and soil [79]. For example, the hybrid species California Mozambique tilapia lives in 44 ppt at the Salton Sea, southern California [80], while another tilapia species (black-chinned tilapia, *Sarotherodon melanotheron*) can tolerate hypersaline environments (∼130 ppt) in an african saline estuary [81]. Species in estuaries and tide pools (which include tilapia species) are exposed to short term salinity fluctuations, and even to vertical or horizontal salinity gradients [76]. For example, the estuarine fish *Fundulus heteroclitus* may be exposed to daily fluctuations in salinity ranging from ∼0.1 ppt to ∼35 ppt [82].

In our study, sub-lethal signs of behavioral and physiological distress, rather than mortality, were chosen to define maximal exposure levels. The NP concentrations used in the present study were chosen based on previously reported experiments, where CuO NP exposure to fish caused toxic effects (see Introduction). Throughout all exposures, only mild signs of distress were observed at the whole organism level, and consistently apparent in a low proportion of the fish in any tank (**Table 1**). We observed a sustained OVR increase for group SW5, prompting us to stop exposure day 8 (**Figure 3B**). As observed, the OVR was affected in a CuO NP concentration-dependent manner in SW, but the response in FW did not show such a trend (although by day 8, we observed an increased OVR at both NP concentrations in FW).

Regulation of OVR is a sympathetic response to stress [83] that can serve to compensate for a reduction in partial O_2_ pressure in blood of fish [84], [85], [86]. Engineered NP have been shown to affect the respiratory system in mammals and, analogously, the gill function and structure in fishes [45], [87], [88]. Moreover, an increase in ventilation rate and respiratory disturbances have been associated with exposure to NP [89], [90]. These earlier findings are consistent with our observation that an increase in OVR is first observed at the highest concentration of NP in combination with additive salinity stress. Interestingly, one common response observed in gills after exposure to different nanomaterials was the production of mucus in trout and zebrafish [87], [90], [91]. Mucus production has been reported as a response to many toxicants for providing a barrier that prevents toxin interaction with epithelial cells [92], [93]. For instance, carbon nanotubes cause respiratory toxicity, increased mucus production, and gill damage in trout [90]. Thus, in addition to inducing oxidative stress, NP may thicken the mucus layer and hinder gas exchange, which might be compensated by elevating OVR. Several studies suggest that the production and composition of gill mucus depends on environmental salinity [94], [95]. Our finding that OVR only increases greatly in SW5, rather than in FW5, supports the notion that gill mucus production depends on environmental salinity and illustrates the importance of the environmental context for assessing the impact of NP.

The effective CuO concentration measured in the tanks was substantially lower over time for all treatments, in particular for SW tanks (**Figure 4A**). However, the relative CuO NP decreased in a similar manner both in presence or absence of fish (**Figure 4B**), showing that the decrease in the NP concentration over time is more likely due to sedimentation and/or adsorption to tank surfaces. This effect is more evident in SW than FW, suggesting that the sedimentation effect might be mediated by the increasing levels of monovalent and divalent cations while salinity was increased during exposure. However, it is important to emphasize that despite of the decrease in the effective concentration to which the fish were exposed particularly in SW, the effects were more marked in SW over (i) the OVR; (ii) intracellular Cu accumulation (higher in both liver and gills in SW0.5 and SW5 compared to SW0 treatment, while in FW the accumulation was observed only at the higher NP concentration, **Figure 8**); and (iii) metal-responsive gene expression. These effects are mediated by the NP still suspended, which did not present a marked increase in their hydrodinamic size. This represents an interesting scenario, where environmental conditions affects effective NP concentration, although the toxic effects of NP are not decreased, but enhanced, supporting the notion that NP toxicity is dependent in the environmental conditions. The measured reduction in NP (Cu) concentration in the tanks cannot be accounted for by the accumulation in fish – the loss of Cu and NPs can only be explained by the unavoidable loss of particles to surfaces in the tank that cannot be quantified directly in any aquatic toxicology study of this sort.

Some considerations can be made to explain the accumulation of intracellular Cu. On one hand, several studies have shown that NP are internalized by cells (even NP with a size of 500 nm). The mechanisms involved in the internalization can be active transport (endocytosis) or passive diffusion through the plasma membrane [13], [96]. For example, hepatic and intestinal human cell lines can uptake Ag and CeO_2_ NP [97]. In trout, hepatocytes can accumulate CeO_2_, TiO_2_ and Ag NP in vitro [98], while when TiO_2_ NP are administered intravenously, they are deposited intracellularly in kidney [99]. Another possibility is that NP could be coated by fish-originated organic compounds (e.g., proteins) or adsorbed in mucus [6], [100], affecting the stability of the NP surface in the vicinity of the exposed tissues, such as gills. Thus, a local release of ions is produced, which are uptaked by the proximal epithelial cells. A similar mechanism has been proposed for toxicity of Ag-NP over zebrafish embryos [101]. It is possible that both internalization of whole NP or uptake of locally dissolved ions can contribute at some extent to the observed accumulation of intracellular copper. To be able of determine which mechanisms are involved in CuO NP uptake under different environmental salinities, more experiments are required to test these hypotheses.

In addition to the effects of salinity stress in combination with CuO NP on the whole organism, two tissues were chosen to analyze the effect of CuO NP on antioxidant mechanisms. Gills are in direct contact with the medium and are critical for osmoregulation (salinity stress compensation) and respiration. The liver is the key organ for detoxification of xenobiotics. Antioxidant mechanisms in these two tissues were analyzed because metal-containing NP have been shown to induce oxidative stress [31]. For instance, in gold fish cells treated with TiO_2_ NP an increase in ROS causing elevated DNA damage was observed [102]. Likewise, exposure of medaka fish (*Oryzias latipes*) to silver NP caused a reduction of SOD activity in liver, as well as a decrease in GSH content in liver, gills and brain [103]. In addition to histological changes in gills, silver NP exposure caused changes in the activity of antioxidant enzymes in gills, liver and skin of *O. mossambicus* [45]. Furthermore, SOD activity in embryos and adult liver and brain was reduced after exposure to iron NP [104]. This change in SOD activity was accompanied by an increase in MDA (malondialdehyde, a measure of lipid peroxidation) and a decrease in GSH levels in brain. Although we did not observe significant changes in SOD activity in gills or liver after CuO NP exposure, CAT and GR activities were significantly affected by CuO NP in both tissues, suggesting that antioxidant mechanisms are also a target for CuO NP (**Figures 5** and **6**). Again, the extent of such responses depended significantly on the environmental context. Additionally, the osmotic stress itself can modify (increase or decrease) the oxidative status in aquatic organisms, at an extent that can be dependent of the species and even tissues [105], [106], [107]. This could explain why CAT activity (gills) was altered by the increase of salinity in our experiments.

Recently, it was shown that in juvenile carp (*Cyprinus carpio*) exposed to ZnO NP, induction of oxidative stress represents a major cause of toxicity associated with these NP [108]. Oxidative stress has also been implicated as the cause of toxicity, structural damage to gills, and mortality in response to CuO NP exposure in zebrafish [87]. Our conclusion that oxidative stress is a major factor in the toxicity of CuO NP in fish is supported by our data on the regulation of antioxidant enzymes. However, we did not observe a large effect of CuO NP on GSH+GSSG, GSSG and GSH. Nevertheless, the ratio of GSH/GSSG was reduced in gills of fish from group FW0.5. It is not clear why this effect was not observed at higher NP concentration (FW5). In any case, the lack of a strong effect of CuO NP on GSH/GSSG ratio suggests that the animals can readily cope with the degree of oxidative stress induced by the NP concentrations used in our study. This high coping ability may be unique to tilapia and a few other species of fish, which are known to be extremely hardy towards many different types of environmental stress [109].

MTs and CYP1A are important proteins for detoxification against metals, and they are coded by metal responsive genes. In our experiments, the mRNA data for *MT* and *CYP1A* in gills (**Figure 7**) agree with the observed regulation of antioxidant enzyme activity and maintenance of the GSH/GSSG ratio. Metallothioneins and other metal-responsive proteins are known to promote the restoration of GSH and antioxidant enzyme activity [110]. In gills and liver, mRNA abundances for *MT* and *CYP1A* increased markedly in response to CuO NP exposure. The increase was not significantly influenced by environmental salinity for *CYP1A* but greatly altered by salinity stress for *MT*. Thus, *MT* mRNA abundance is regulated differently in response to CuO NP depending on the particular environmental context. The increases of either *CYP1A* and particularly *MT* mRNA abundance generally correlate well with increment in the intracellular Cu concentration in gills and liver (an increase in the intracellular concentration of metal induces the expression of one or both of them). Metal transcription factors (MTFs) recognize the MRE in DNA and activate the transcription of metal responsive genes, and have been characterized in different species, including Mozambique tilapia [111]. MTF translocates from cytoplasm to nucleus in response to the increment of heavy metals (Zn^2+^, Cd^2+^, Cu^2+^, etc), but given that MTFs can only directly bind Zn^2+^, the molecular mechanisms involved in this activation for other metals are no yet fully understood, although different MTF isoforms might be required [111], [112]. Two possible pathways have been proposed for MT-induction by Ag NP in astrocytes [113]: (i) dissolved Ag^+^ ions from NP compete and displace Zn^2+^ from the MT-Zn^2+^ pools, causing an activation of MTF-mediated MT gene expression, and (ii) oxygen radicals generated at the NP surface induce MT expression via activation of the Nrf1 transcription factor. Cellular uptake of NP could be achieved by either endocytosis, with a concomitant vesicular transport through the cell or the fusion of vesicles with lysosome [21], or through diffusion, where NP can be deposited in other organelles, such as mitochondria and the nucleus [114]. Differences in the pH of these organelles or coating by biomolecules may alter the stability of NP [31], releasing ions from their surface which could result in induction of the metal-responsive genes.

In summary, the present study shows that manufactured CuO NP can trigger sub-lethal responses associated with oxidative stress in Mozambique tilapia. Such responses are discernible at the whole organism level (OVR increase) as well as at the molecular level in gills and liver (antioxidant enzyme activity, GSH/GSSG ratio, metal-binding protein mRNA abundance). Importantly, we show that responses to CuO NP exposure depend greatly on the environmental context, i.e. whether fish are exposed in freshwater or while facing additive salinity stress. The uptake of CuO NP may be affected by such environmental context – exposure routes could differ in fish exposed at different salinities, as well as the availability of NP. As aforementioned, it is well known that fish drink large amounts of seawater to compensate for diffusional loss. Therefore, intestinal uptake of NP may preferentially occur in high salinity environments but may be negligible in freshwater. Thus, it is critical to carefully assess the environmental context-dependence in studies evaluating the ecological relevance of the potential toxicity of manufactured NP.

## Supporting Information

File S1 Includes the following files: **Figure S1.** Schematic of flame generated NP synthesis system and collection in a baghouse filter. **Figure S2.** XRD pattern of the CuO NP. (**A**) XRD of the as-synthesized NP (**B**) XRD of the CuO powder samples probe sonicated for 5 min with a 20 s sonication followed by 10 s rest. Both the patterns are identical suggesting no change in physicochemical properties due to sonication. **Figure S3.** The Zeta Potential of the NP dispersion in fresh water fish tanks at different time points (from top to bottom, Day 0 to Day 8). **Table S1.** Data from CuO NP characterization. ^a^Solvent: the medium in which NP were suspended (FW freshwater, SW increasing salinity, 5 and 0.5 represent the NP concentration in the sample, in mg·L^−1^). ^b^Time (in days). T = 0 represents the time immediately after NP were added to the medium. ^c^Values: mean ± *SD*.
(DOC)
