# Supplementary material for: Sublethal Effects of CuO Nanoparticles on Mozambique Tilapia (Oreochromis mossambicus) Are Modulated by Environmental Salinity
Source: PLoS One. 2014 Feb 10;9(2):e88723. doi: 10.1371/journal.pone.0088723 (PMC3919801; doi:10.1371/journal.pone.0088723)

**Supplementary Information**

**Sublethal effects of CuO nanoparticles on Mozambique tilapia (*Oreochromis mossambicus*) are modulated by environmental salinity**

Fernando Villarreal^1^*^¶^, Gautom Kumar Das^2¶^, Aamir Abid^2^, Ian M. Kennedy^2^ and Dietmar Kültz^1^

^¶^ Authors contributed equally to this work

^1^Physiological Genomics Group, Department of Animal Science, University of California-Davis

^2^Department of Mechanical and Aerospace Engineering, University of California, Davis, CA 95616, USA

* Corresponding author

e-mail: [fvillarreal@ucdavis.edu](mailto:fvillarreal@ucdavis.edu)

**Figure S1**. Schematic of flame generated NP synthesis system and collection in a baghouse filter.

**Figure S2.** XRD pattern of the CuO NP. **(A)** XRD of the as-synthesized NP **(B)** XRD of the CuO powder samples probe sonicated for 5 min with a 20 s sonication followed by 10 s rest. Both the patterns are identical suggesting no change in physicochemical properties due to sonication.

**Figure S3.** The Zeta Potential of the NP dispersion in fresh water fish tanks at different time points (from top to bottom, Day 0 to Day 8).


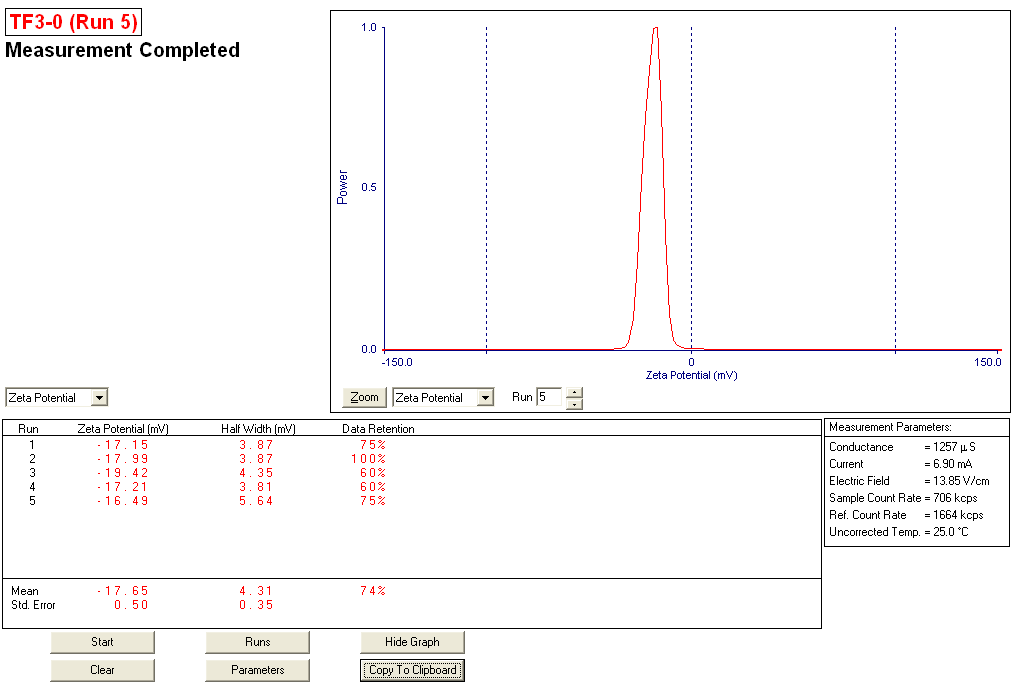


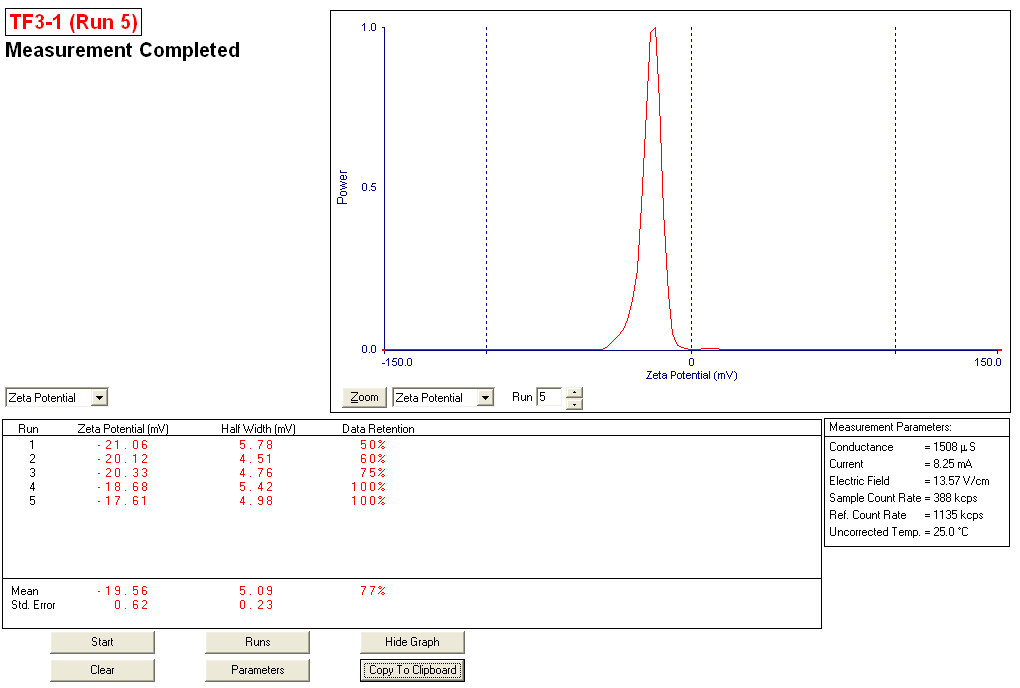


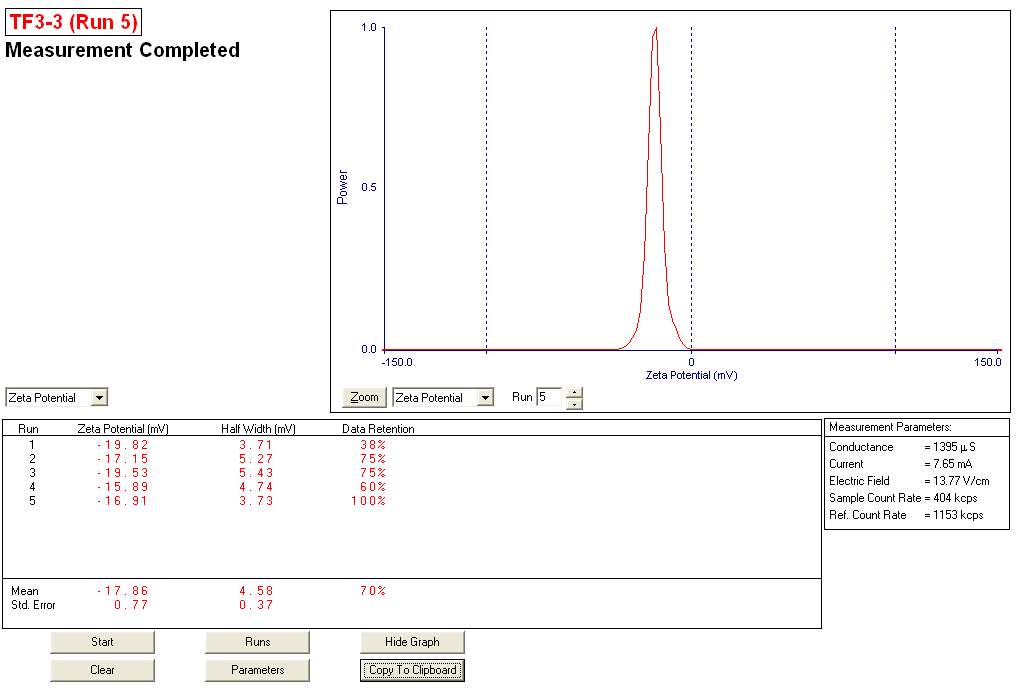


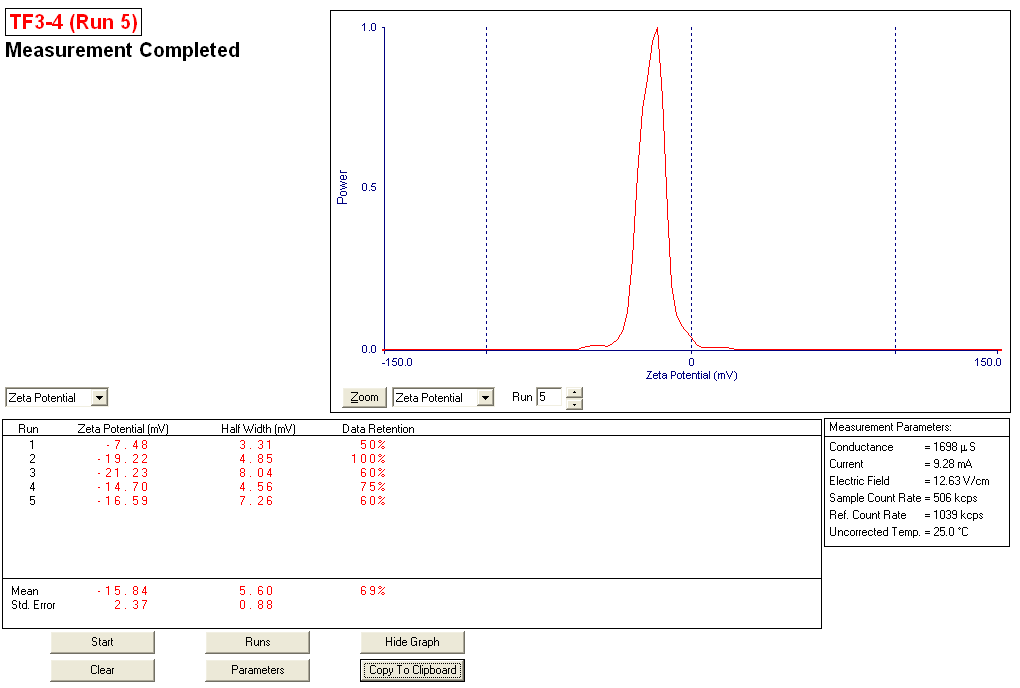


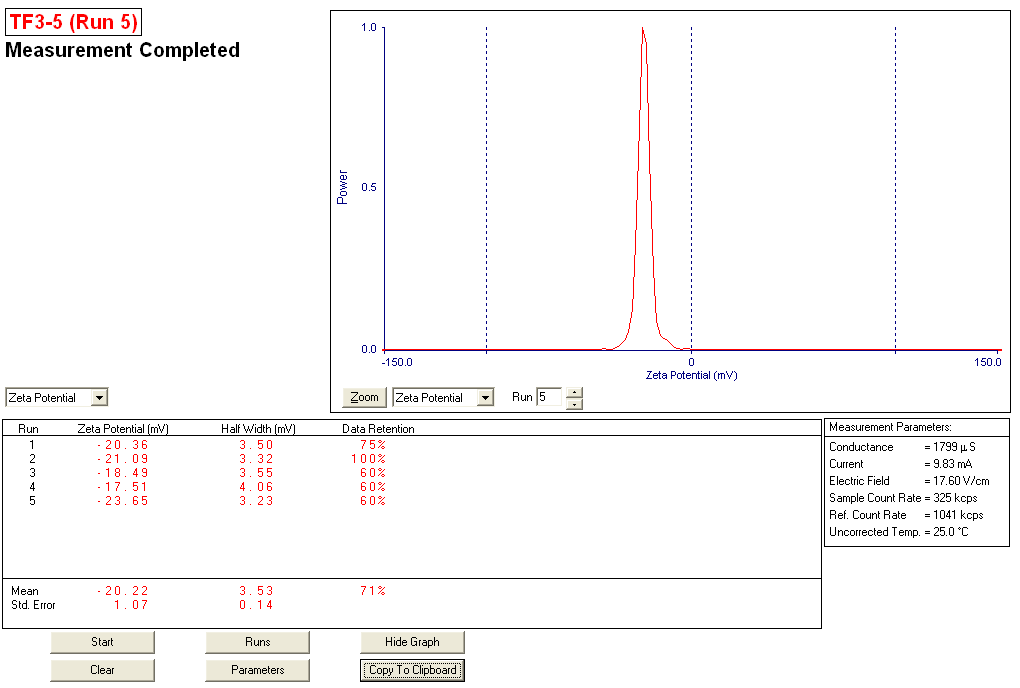


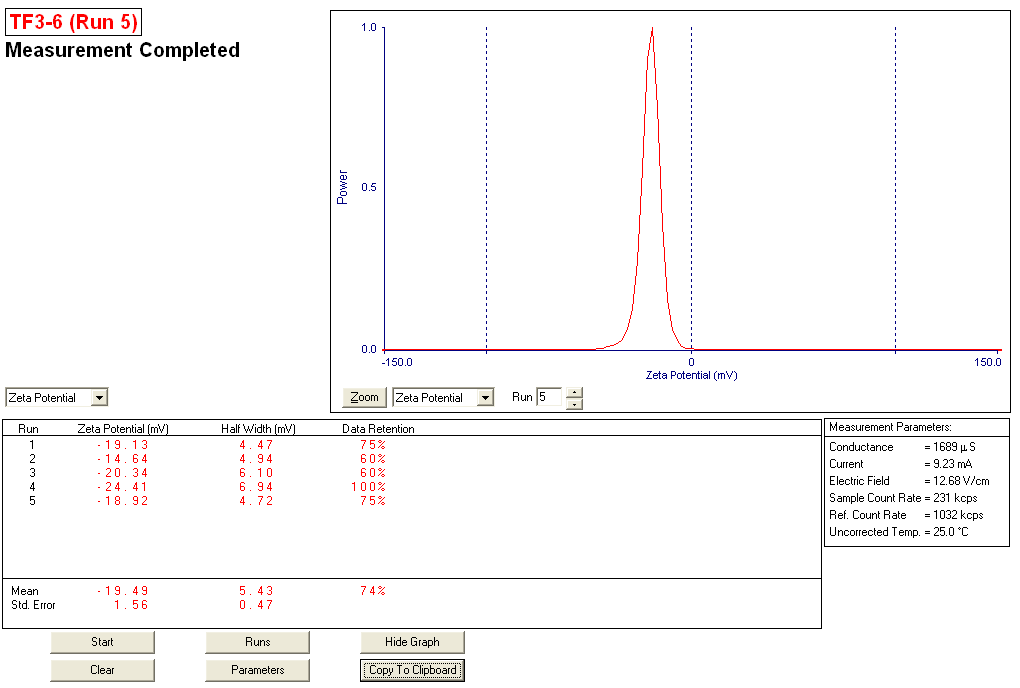


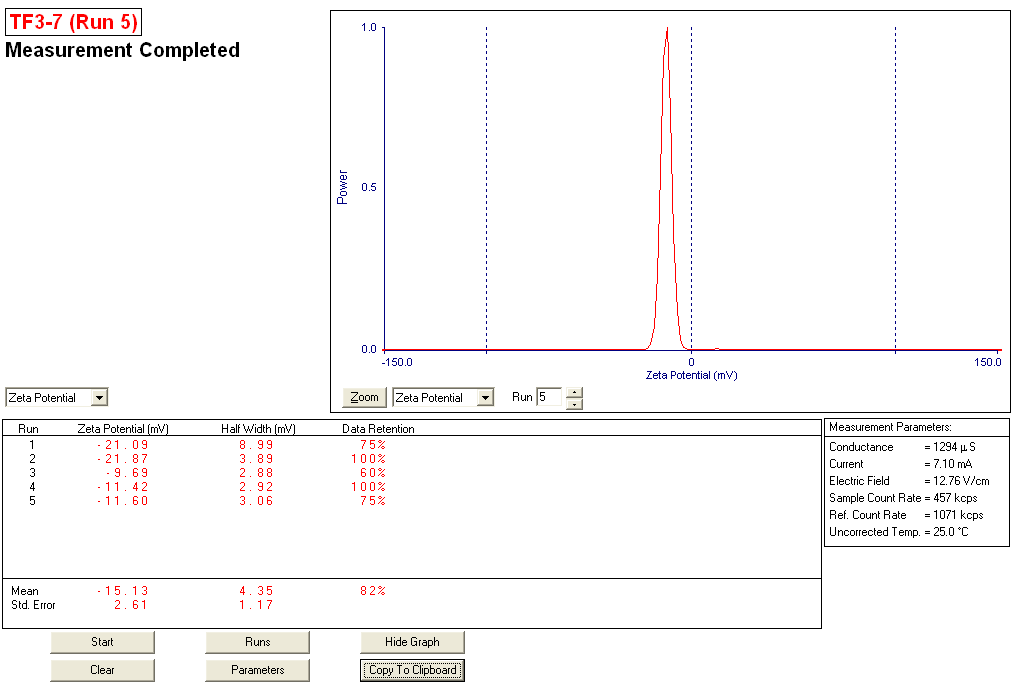


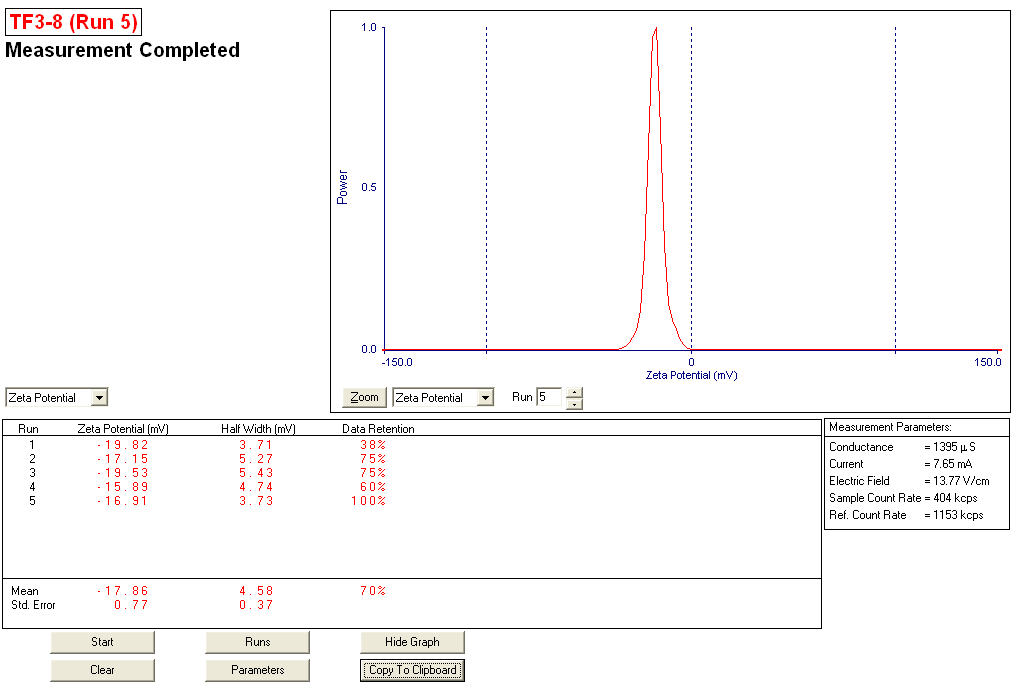

Supplement: File S1 — Includes the following files: Figure S1. Schematic of flame generated NP synthesis system and collection in a baghouse filter. Figure S2. XRD pattern of the CuO NP. (A) XRD of the as-synthesized NP (B) XRD of the CuO powder samples probe sonicated for 5 min with a 20 s sonication followed by 10 s rest. Both the patterns are identical suggesting no change in physicochemical properties due to sonication. Figure S3. The Zeta Potential of the NP dispersion in fresh water fish tanks at different time points (from top to bottom, Day 0 to Day 8). Table S1. Data from CuO NP characterization. aSolvent: the medium in which NP were suspended (FW freshwater, SW increasing salinity, 5 and 0.5 represent the NP concentration in the sample, in mg·L−1). bTime (in days). T = 0 represents the time immediately after NP were added to the medium. cValues: mean ± SD. (DOC) [file pone.0088723.s001.doc]
